# Foresight in public health: a tutorial on application and insights on challenges from the PHIRI foresight exercise

**DOI:** 10.1093/eurpub/ckae040

**Published:** 2024-07-01

**Authors:** Elizabeth N Mutubuki, Daniela Moye-Holz, Mariana Peyroteo, Luís Velez Lapão, Henk B M Hilderink

**Affiliations:** Centre for Public Health, Health Services and Society, National Institute for Public Health and the Environment (RIVM), Bilthoven, The Netherlands; Centre for Public Health, Health Services and Society, National Institute for Public Health and the Environment (RIVM), Bilthoven, The Netherlands; UNIDEMI, Department of Mechanical and Industrial Engineering, NOVA School of Science and Technology, Universidade NOVA de Lisboa, Caparica, Portugal; Laboratório Associado de Sistemas Inteligentes, LASI, Guimarães, Portugal; UNIDEMI, Department of Mechanical and Industrial Engineering, NOVA School of Science and Technology, Universidade NOVA de Lisboa, Caparica, Portugal; Laboratório Associado de Sistemas Inteligentes, LASI, Guimarães, Portugal; WHO Collaborating Center on Health Workforce Policy and Planning, Instituto de Higiene e Medicina Tropical, Universidade NOVA de Lisboa, Lisbon, Portugal; Centre for Public Health, Health Services and Society, National Institute for Public Health and the Environment (RIVM), Bilthoven, The Netherlands

## Abstract

**Background:**

The application of foresight to the field of public health is limited. There is growing need to anticipate uncertain future trends and to plan for them. Foresight provides tools to experts and policymakers to discuss and plan for possible futures. Hence, the aim of this study is to illustrate how the foresight six-step approach can be applied in public health, and to provide recommendations on dealing with challenges, drawn from the Population Health Information Research Infrastructure (PHIRI) foresight exercise.

**Methods:**

In this tutorial, we describe the six-step approach as part of foresight methodology and give examples of possible challenges. Step 1 comprises the formulation of study objectives. Step 2 focuses on developing a conceptual model and applying the Demographic Economic Sociocultural Technological Ecological and Political-Institutional (DESTEP) framework to identify and prioritize driving forces for the topic of interest. In Step 3, a time horizon and spatial level are defined. Step 4 discusses scenario logics. Steps 5 and 6 discuss different types of scenarios and associated tools for analyses. Possible challenges encountered whilst applying the foresight methodology at each of the steps, were drawn from experiences during PHIRI foresight exercise.

**Results:**

Challenges associated with applying the foresight six-step approach included: formulating concise objectives, developing a conceptual model, understanding driving forces and uncertainty and difficulties in building scenarios.

**Conclusions:**

Understanding concepts used in the six-step approach and how they relate to each other remained difficult. Support from foresight experts, conducting more foresight exercises, tutorials and guidelines can enhance understanding and support building capacity.

## Introduction

The increase in the number of publications in the field of strategic foresight, as well as the attention to foresight by countries, international organizations and public health conferences, is an indication of growing interest in this field.^1^ Countries such as, the United Kingdom,^2^ Finland,^3^ Australia,^4^ Singapore^5^ and the Netherlands^6^ apply foresight for decision making within public health. In the Netherlands, for example, foresight studies are used to provide input for the National Health Policy Memorandum of the Ministry of Health.^7^ The use of foresight studies within public health, however, remains limited compared to other fields, such as the environmental field,^7^ which has a well-established history of conducting scenario studies on climate change.^8^ Reasons for the limited number of public health foresight studies (PHFS) include the complex multi-causal nature, the role of human behaviour in health outcomes, limitation in resources, a reactive rather than proactive approach in public health, and limitations in understanding underlying mechanisms.^9^^,^^10^

In recent years, the definition of foresight has become more comprehensive, with an emphasis on foresight being an exploratory, participatory, action-oriented and visionary activity.^11^ Miles (2003) describes foresight as the ‘application of systematic, participatory, future-intelligence gathering and medium-to-long-term vision-building processes to informing present-day decisions and mobilizing actions’.^12^ The participatory nature of foresight is beneficial for decision-making processes because it results in new intelligence, acquired from an increased awareness of the different ideas that various stakeholders hold regarding the future.^13^ In addition, it promotes shared visions of challenges and opportunities, thereby enabling consensus in a diverse political landscape^13^ and the development of appropriate and robust policies.^14^

Foresight consists of processes and methodologies, which provide a systematic and efficient way to address possible futures.^15^ Processes are the steps taken to explore possible futures and include framing the issue, communication, stakeholder mapping and participation*.* Foresight methodology focuses on the tools and methods used during the foresight processes to conduct and structure a study, collect and analyze data to create insights regarding the future.

Foresight is plural in nature, and this complexity of foresight brings challenges when conducting a foresight study, such as which methods to use and when. Various foresight methods and processes exist and the choice of which depends on the issue, context, available resources, and purpose of given foresight studies. Most used methods, such as the two axis-approach^16^ focus on only two main trends, whereas a diversity of relevant trends that might impact the topic of study exist. Using only two main trends does not fully represent the uncertainty of future societal challenges that we might face. Whereas the foresight six-step approach is a diverse approach that takes into consideration the diversity of relevant trends. Therefore, in this paper, we aim to provide guidance on how to conduct a foresight study using the six-step foresight approach, and provide recommendations on how to deal with associated methodological considerations, drawing on experiences from the Population Health Information Research Infrastructure (PHIRI) project.

The main aim of the PHIRI project was to facilitate and generate evidence for research regarding the health and wellbeing of populations impacted by COVID-19.^17^ Within the PHIRI project, the PHIRI foresight exercise was conducted amongst European Union (EU) Member States (MS). The PHIRI foresight exercise consisted of a capacity building course and application of the foresight capacity built. In total, 11 PHFS were conducted. Participants were supported in planning their own PHFS using the six-step foresight approach. During this process, insights on the challenges they experienced were gained and so were lessons learnt for future improvements. The National Institute for Public health and the Environment (RIVM) led the PHIRI foresight exercise, building upon its more than 30 years of experience in PHFS. The RIVM and other partners formed the project team for PHIRI foresight exercise.

## Methods

### The six-step foresight approach

**Figure 1:**
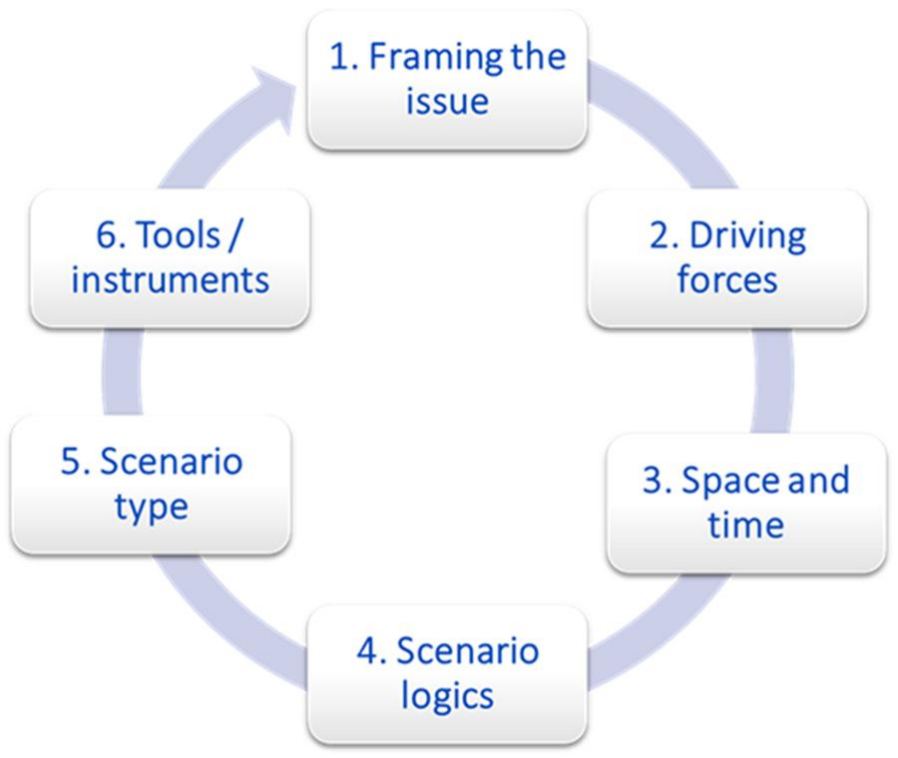
The six-step foresight approach^21^^,^^22^

A stepwise procedure as part of a foresight study is common, and there are various approaches for this.^18–20^ Derived from these existing approaches, RIVM developed a six-step foresight approach (figure 1), which covers the most essential steps to systematically develop scenarios. This iterative approach has been applied to public health topics several times and proved to be useful.^7^ The six-step approach is conducted as part of a broader foresight process, which comprises aspects like having a governance structure of the project, communication plan, stakeholder mapping and participation. In this paper, we focus on the scenario development resulting from the six-step approach. This is because scenario development forms the core of foresight. However other aspects such as processes and participation are also essential in doing a foresight study.

### Step 1: selection and framing of the issue

The first step in the six-step foresight approach (figure 1), is selection of the issue that will determine the objective of the study. In this step, the research question, main issues, and sub issues should be identified. Here, it is important to answer the question, ‘why is the foresight study being conducted, and why are the scenarios being built’. A clearly defined research question or objective will enable a clear structure and direction of the study. Research questions in foresight studies often have particular focus on uncertainties (e.g. possible future trends in overweight), policy (e.g. possible strategies to achieve policy targets for overweight, given the uncertainties), participation (e.g. engaging actors involved in tackling overweight), or a combination of these three. The Dutch PHFS, for example, adopts the following research questions: what are the most important future trends for public health and health care, which societal challenges arise from these trends, and what can be done to target these challenges.

### Step 2: conceptual model and driving forces

#### Conceptual model

**Figure 2:**
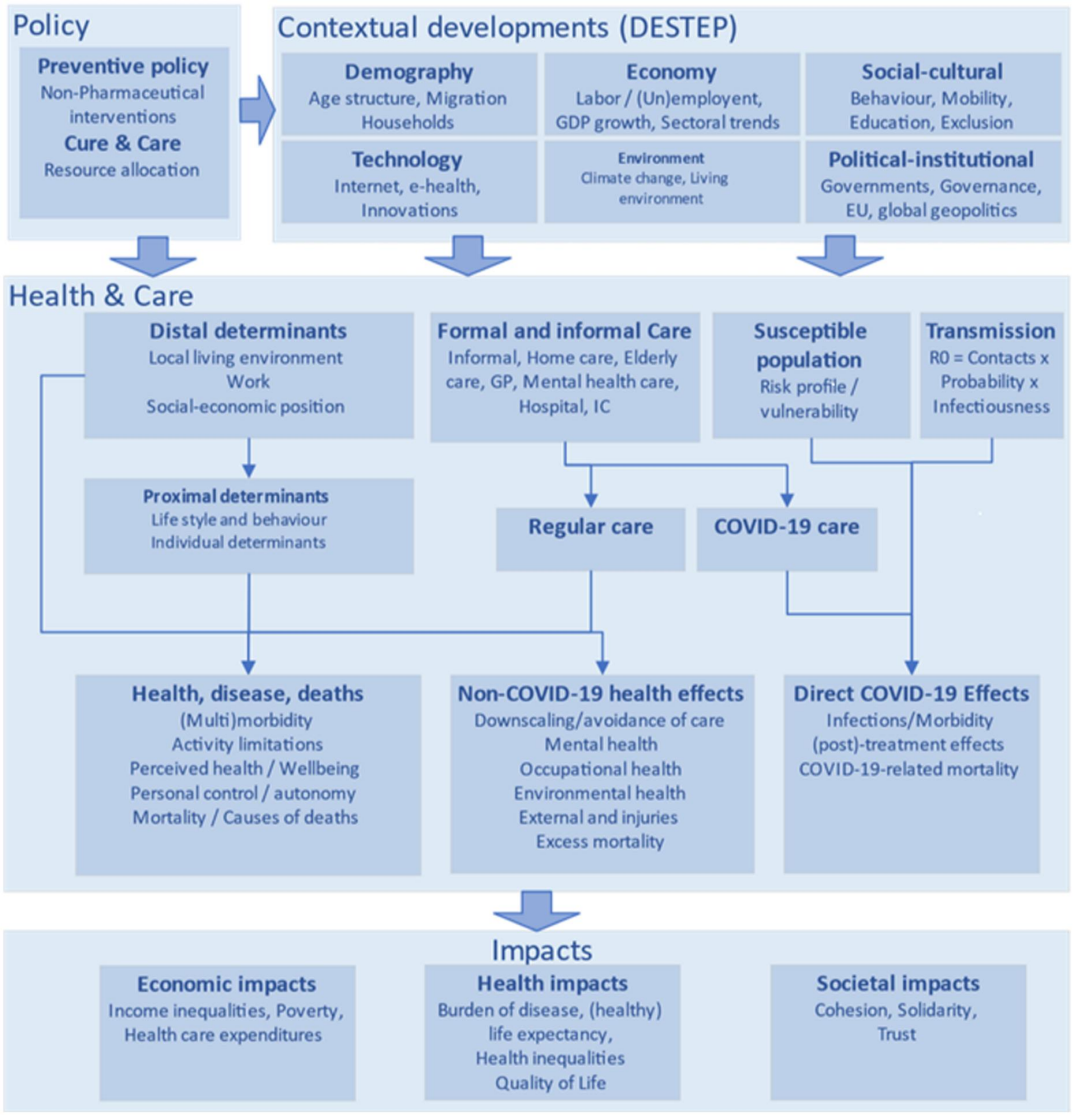
Conceptual model used to understand the wider impacts of the COVID-19 pandemic^24^

A conceptual model in a foresight study is used to indicate interactions between a given topic and other aspects of influence.^23^ It serves to structure ideas, relevant aspects of a given topic of study and provides a roadmap or framework for the study. Figure 2 shows an example of a conceptual model, which is used in the Dutch PHFS, including the Dutch COVID-19 PHFS and in PHIRI foresight exercise.^23^

#### Indicators

Indicators [e.g. disability-adjusted life years (DALYs), life expectancy, prevalence of smoking] are used and linked to a conceptual model to make the model more concrete. They provide ‘operationalization and quantification of the conceptual model’.^23^ Indicators can be organized differently, such as categorizing indicators into inputs and processes (e.g. governance), outputs (e.g. prevention programmes), outcomes (risk factors), and impacts (health status). Indicators should be accompanied by clear and structured data. Clear and structured data will ensure credibility, comparability, transparency, and reproducibility of PHFS. An example of a clear and structured data source is the Health Information Portal.^25^

#### Identifying driving forces

Identifying driving forces refers to identifying trends that can have influence on the processes, outcomes and topic of study. To identify these driving forces, the Demographic Economy Socio-cultural Technological Ecology and Political-Institutional (DESTEP) framework can be used. DESTEP provides a broad inventory of external factors that may have an impact on the topic of study. There are also similar categorizations such as Political Economic Social Technological Legal and Environment (PESTLE) and Social Technological Economic Environmental Political Legal Ethical Demographics (STEEPLED), where legal and ethical trends are also considered. The choice of which categorization to use depends on the issue at hand, which determines the relevance of other categories. For most public health issues, DESTEP has proven to be adequate.^7^^,^^23^

#### Ranking driving forces

To select the most important trends to be considered, driving forces should be ranked according to relevance and uncertainty. Identification of relevant and uncertain trends can be done using results from the DESTEP exercise, through a desktop research and/or stakeholder consultations and workshops. Ranking driving forces in terms of relevance refers to how much impact the driving forces may have on the outcomes and the topic of study. Ranking driving forces in terms of uncertainty can encompass the likelihood of occurrence in combination with uncertain impacts on outcomes. This way of ranking uncertainties with limited knowledge can be referred to as cognitive uncertainty. In addition, normative uncertainties can also be distinguished. These refer to how people value outcomes and trends and consequently rank driving forces differently. For example, some people might find increasing health care expenditures one of the most important outcomes, while others might find health inequalities most important. It is important to be aware of the difference between cognitive uncertainties (referring to limited knowledge) and normative uncertainties (referring to what people value most).

### Step 3: spatial unit and time

In this step, the time horizon for looking into the future is set depending on the issue at hand. For example, looking at the long-term health impacts of a smokefree generation might require a time horizon further in time, than when looking at the indirect health impacts of the COVID-19 pandemic. In addition, the spatial unit is defined as geographical coverage. That is, whether the PHFS will be conducted at an international, national, regional or local level.

### Step 4: scenario logics

By analyzing the main trends and driving forces identified using the DESTEP (Step 2), the choice for certain scenario logics can be made. Based on the number of identified main uncertain trends, one can follow different scenario logics. For example, with one main uncertain trend the choice can be made for high-low or best/worst case scenarios.^26^ If there are two main uncertain trends, they can be combined into a two-axis approach resulting in four scenarios (i.e. the four quadrants of the two axis). When there are more than two uncertain trends, the combination of these trends can result in several possible scenarios. In this case, a morphological-mixing panel approach can be applied.^27^ The resulting set of scenarios can range from two to even more than five. In addition to these options, there are different variations possible. For example, high-low scenarios can be expanded with a medium scenario. It is also possible to choose for one scenario, for example a trend or business-as-usual scenario that can be used as a reference for, for example, policy variants.

### Step 5: types of scenarios

Depending on the scenario logics and aims, scenarios can be quantitative, qualitative or a combination of both. Most foresight studies include both, and therefore apply both quantitative and qualitative methods.^28^ Quantitative scenarios involve analysis of quantitative data by describing future scenarios using quantitative figures. Incorporating specific quantitative results regarding critical future trends or outcomes may enable a heightened awareness of decision makers. Qualitative scenarios can be for example, scenario storylines based on qualitative data. For these data, the inputs of different stakeholders may helpful or even necessary.

### Step 6: tools and instruments

A number of tools and instruments can be used to support both the qualitative and quantitative facets of scenarios. Choice of tools to use depends on the purpose, availability and applicability of the tool. Examples of qualitative tools include opinion surveys, experts’ interviews, focus groups, morphological analysis, historical analogy and visioning. Whereas examples of quantitative tools include probabilistic forecasting, stochastic processes analysis, regression analysis and epidemiological models. Preferably, most aspects of the quantitative scenario are generated by mathematical simulation models that describe the epidemiological dynamics. This enables consistency between results for example, regarding projections of risk factors and health outcomes. However, in public heath, only few of these models are available; DYNAMO HIA,^29^^,^^30^ RIVM Chronic Disease Model.^31^ Regression analysis and trend extrapolation are applied to quantify the scenarios (see e.g. Methods VTV-2018).^7^

## Results

### Challenges experienced in the application of foresight and suggestions on how to deal with them

Although the six-step foresight approach provides a clear and a systematic way of conducting PHFS, our experience during PHIRI foresight capacity building course was that, participants still experienced challenges in planning and developing their respective PHFS. The participants were asked to provide feedback on these challenges using summary sheet forms. The challenges described in this paper are both the experiences of the participants and those of us (PHIRI project team-organizers of the foresight capacity building course). Most participants experienced challenges in developing a conceptual model and understanding the driving forces. Another commonly experienced challenge was building the scenarios due to the inability to take into consideration the interaction between driving forces and the outcomes. A more detailed description of the PHIRI foresight capacity building course can be read elsewhere.^21^Table 1 lists the challenges that were encountered at each stage of the six-step foresight approach while conducting a PHFS, and recommendations on how to deal with these challenges.

**Table 1:** Challenges experienced whilst applying the six-step foresight approach in the PHIRI foresight capacity building course and the associated recommendations

Six-step approach	Challenges	Recommendation on how to deal with challenges
*Step* 1: selection and framing of the issue	Formulating clear research questions and the associated objectives.	A clear research question should be stipulated before moving on to the next step. Narrow down from general issues to more specific sub-issues.
*Step* 2: conceptual model and driving forces	Developing a concise and simple conceptual model with a clear roadmap, considering relevant inputs and outcomes, and identifying related indicators to these components. Understanding that driving forces refer to trends, which are assumed developments in the future. Driving forces are not variables. Identifying the trends to be considered in the study by choosing those that are most important and most uncertain. Understanding the concept of uncertainty in the context of foresight.	Use existing conceptual models for inspiration. Keep the conceptual model simple, clear and concise. Use concrete and (preferably) existing indicators with data available. If needed, develop proxy indicators. Understanding what a trend entail. Use a systematic approach such as looking into the literature for relevant trends influencing a topic Following the DESTEP exercise to identify major trends. The DESTEP exercise can support the identification of these trends by ranking them by relevance (major impact on the topic) and uncertainty (most unlikely or least information about such trend). Uncertainty in foresight refers to the level of knowledge or likelihood of a trend. This concept needs to be thoroughly explained and understood by the researchers and stakeholders involved.
*Step* 3: spatial and time	Considering the complexity between doing a national or a regional study. Considering that the foresight study covers years into the future (e.g. 5–10 years after the completion of the study).	During the planning phase, consider if there is access to data, resources and stakeholders at the national or regional level. This could determine the feasibility of the study. Understand that foresight explores the future and should consider several years into the future.
*Step* 4: scenario logistics-main uncertainties	Based on the number of identified uncertain trends, follow a scenario logic that takes into consideration and relates all the chosen driving forces.	Consider how one or more uncertain (and certain) trends can influence each other into the future. Consider all trends into one plausible scenario.
*Step* 5: types of scenarios	Balancing the input and interests of stakeholders and team members when building the scenario storylines. Making assumptions about the future. The use of quantitative tools.	Building the scenario storyline requires a qualitative approach through stakeholder participation. It also requires a certain level of creativity, imagination and assumption to build such stories for each scenario. Quantitative tools (e.g. modelling, forecasting) can be used when possible, to enrich the stories of the scenarios. If there is capacity (within the team) to build such models or forecast, or if these are already available from other sources, it is recommended to quantify and showcase projections and changes of trends.
*Step* 6: tools and instruments	Identifying tools that can be used to build scenarios.	Qualitative tools include stakeholder participation and consultation to build scenarios depending on the driving forces and their interactions. Quantitative tools include those already existing (e.g. projections of trends). It is also recommended to have the necessary capacity within the team in modelling and forecasting.

Note: Challenges experienced while applying the six-step foresight approach were drawn for the PHIRI foresight capacity building course.^21^

#### Step 1 challenges: formulating clear research questions and associated objectives

Narrowing down from a general topic to clear research objectives can be challenging. Knowledge and understanding of a given topic including related priorities is of importance when defining research questions and prioritizing issues to address. Not all issues can be addressed in one PHFS. Clear and specific objectives allow for a manageable and concrete study. A clear research question should be stipulated before moving on to the next step. However, given that the six-step approach is an iterative process, research questions can be revised and adapted if needed.

#### Step 2 challenges: developing a conceptual model and understanding the driving forces

Selecting and developing a conceptual model, defining driving forces and associated indicators can be problematic. Using and adapting already existing conceptual models is a useful way to begin. Developing a conceptual model requires intensive consideration of all aspects influencing the outcomes of study. Identifying appropriate indicators can be done by engaging stakeholders with relevant expertise. A practical choice of indicators is recommended, in the absence of indicators proxy indicators can be used. Challenges in defining driving forces can be rooted in misunderstanding of the concepts. A common misunderstanding was that driving forces refer to variables instead of trends. To identify and rank driving forces, it is important to understand first what a trend entails and what foresight refers to as important and uncertain. A trend is an assumed development in the future with long-term and lasting effects. Identifying trends can follow a systematic approach starting with literature review followed by stakeholder consultations. During the ranking process, defining first what one could consider important and uncertain can support an efficient ranking process.

#### Step 3 challenges: complexities in defining time horizon and geographical extent

Consideration of complexities associated with completing a PHFS at a certain geographic level is often ignored. Practicalities such as availability and access to data and resources should be considered during the planning phase, as these influence feasibility and implementation of given PHFS. Foresight explores the future and should consider several years into the future. A period of more than 5 years into the future (after the completion of the PHFS) is recommended and can provide interesting insights.

#### Steps 4, 5 and 6 challenges: developing scenarios

Steps 4, 5 and 6 entail the structuring and actual development of scenarios. A number of participants experienced difficulties with developing scenarios. Identifying the most uncertain trends helps to determine the nature of the scenarios (scenario logics), and their relationship with each other and other relevant trends. Understanding this relationship will enable the actual development of scenarios and their characteristics. Constructing scenarios requires input from relevant stakeholders, a certain level of assumption on how the future could look like, and the use of tools appropriate to the context of the study. The type of scenarios can also be determined by the capacity and motivation of the team, the input from stakeholders, and data available. The use of both qualitative and quantitative data is recommended but considerations of their availability, actual use and analysis may limit or enrich the development of scenarios.

## Discussion

There is a clear opportunity to improve the use of foresight in public health. The COVID-19 pandemic showed how important foresight can be to support decision-makers in dealing and preparing for uncertain futures. More public health actors recognize this importance.^32^^,^^33^ There is no blueprint for doing a PHFS. The six-step approach covers most common elements of foresight, such as horizon scanning, trend analysis and scenario development.

### Our findings regarding the challenges experienced during applying the six-step approach

Our findings indicate that formulating clear research questions, developing a conceptual model and understanding the driving forces, defining time horizon and geographical extents, and developing scenarios are challenges that can be encountered while applying the six-step foresight approach when planning and developing a PHFS.

### Strengths and limitations

To the best of our knowledge, the current paper is the first tutorial paper to report step-by-step a foresight methodology: the six-step foresight approach in the public health domain. A number of foresight studies are reported through grey literature and in most cases the methods or processes taken cannot be reproduced. Foresight protocols mention what should be done to conduct a foresight study, but do not explain how to conduct it.^34^^,^^35^ The six-step foresight approach explained in this paper is a reflection of approaches from grey literature. It is a stepwise approach that allows for a systematic, reproducible, and transparent approach towards conducting foresight studies. In addition, the six-step approach is an iterative process that allows continuous building and refining of a given topic, but demands time, financial resources, and human capacity, all of which may be scarce.

Another strength of the six-step foresight approach is its diverse approach for scenario development, which takes into account the diversity of relevant trends. This is important because the future is shaped by a multitude of factors. Focusing on one or two trends can lead to a narrow and biased understanding of the future. Another strength of the six-step foresight approach is its generic nature, which allows it to provide guidance on how to conduct foresight studies across disciplines. At each step choices for different methods that are applicable to a given topic of study can be made. Other foresight approaches have been too specific, limiting their use across disciplines. The six-step foresight approach is participative in nature, hence allows for diversity in perspectives regarding the future and understanding of what consequences they might have for policy and decision making. This is important because it enables development of policies that are both robust and representative. However, to achieve this, the influences of the diverse stakeholders should be adequately managed. This is challenging in practice because, applying a stakeholder mapping process that addresses these aspects requires time, resources, and a balanced representation of different stakeholder groups that have interest in the topic of study.

Developing a foresight study may entail more complexities and necessitate further deliberations despite the suggested methodological approach outlined in this article. Hence, the six-step foresight approach is a guide and not a manual on how to conduct a PHFS. Another limitation is that, understanding some of the concepts of six-step foresight approach (i.e. scenario building and making assumptions about the future that are not always based on data) requires a paradigm shift in the way of thinking and experience. For example, all existing scientific evidence is about the past, while we want to support policy making in the future. However with practice, and a certain level of imagination and creativity in thinking about the future we want in public health can make this possible.

### Implications for research and practice

One can argue that there are already various foresight approaches available. This paper acknowledges that, and places emphasis on the need for a stepwise approach in the application of the available methods, in order to enable reproducibility and transparency in conducting PHFS. In addition, our experience from the PHIRI foresight capacity building course showed that there is need for continuous capacity building, guidance, and support from experts when conducting a PHFS. Therefore, capacity-building programs and clearly defined approaches can provide useful knowledge to overcome these challenges. Foresight approaches such as the six-step approach, may also be useful in other disciplines, such shaping research and development for global public health.

## Conclusion

The six-step approach, though its applications by RIVM and in the PHIRI project has proven to be a useful tool in conducting PHFS. We recognize that the six-step approach is not a manual but a guide on how to conduct a PHFS. This is a field in continuous growth and development with valuable insights for decision making. In order to expand and exploit the use of foresight in public health, building more capacity in foresight is needed.

## Data Availability

No new data were generated or analyzed in support of this research. Key points Foresight is a valuable and underused tool to explore possible futures for policy-making support. Although the six-step approach in foresight is a useful tool, its implementation comes with challenges. Further capacity building and sharing of experiences are necessary to advance the field of foresight in public health.
